# R&D in Vaccines Targeting Neglected Diseases: An Exploratory Case Study Considering Funding for Preventive Tuberculosis Vaccine Development from 2007 to 2014

**DOI:** 10.1155/2017/4765719

**Published:** 2017-01-04

**Authors:** Theolis Costa Barbosa Bessa, Erika Santos de Aragão, Jane Mary Medeiros Guimarães, Bethânia de Araújo Almeida

**Affiliations:** ^1^Gonçalo Moniz Institute, Oswaldo Cruz Foundation Unit in Bahia State, Rio de Janeiro, RJ, Brazil; ^2^Institute of Collective Health, Federal University of Bahia, Salvador, BA, Brazil; ^3^Institute of Humanities Arts and Sciences, Federal University of Southern Bahia, Itabuna, BA, Brazil

## Abstract

Based on an exploratory case study regarding the types of institutions funding the research and development to obtain new tuberculosis vaccines, this article intends to provoke discussion regarding the provision of new vaccines targeting neglected disease. Although our findings and discussion are mainly relevant to the case presented here, some aspects are more generally applicable, especially regarding the dynamics of development in vaccines to prevent neglected diseases. Taking into account the dynamics of innovation currently seen at work in the vaccine sector, a highly concentrated market dominated by few multinational pharmaceutical companies, we feel that global PDP models can play an important role throughout the vaccine development cycle. In addition, the authors call attention to issues surrounding the coordination of actors and resources in the research, development, manufacturing, and distribution processes of vaccine products arising from PDP involvement.

## 1. Introduction 

Vaccination is considered one of the main public health strategies to prevent, control, and eradicate a number of infectious diseases, due to cost-effectiveness in comparison to other types of health measures. Immunization strategies have proven effective in reducing the incidence of disease among vaccinated individuals, and when high vaccination rates are reached in populations, disease transmission may be significantly impacted, resulting in both individual and collective protection [1, 2]. A recent study has shown that each dollar invested in vaccination programs results in a $16 savings in health care expenditures, lost wages, and lost productivity due to illness [3].

In spite of this, it has been estimated that 1.5 million children die worldwide each year from diseases otherwise preventable by vaccination [4]. In 2014, 18.7 million children around the world did not receive a basic schedule of vaccines [5]. To minimize bottlenecks in access to existing vaccines destined to the pediatric segment, the Global Alliance for Vaccines and Immunization (GAVI) was created in 2000 to obtain and distribute cheaper vaccines to the world's poorest countries.

Despite receiving vaccines at lower prices, the GAVI-eligible countries still experience difficulties in guaranteeing the allocation of resources to purchase essential vaccines. Moreover, even ensuring the bare minimum conditions of properly storing vaccines at low temperatures is not always feasible in remote areas. It is therefore necessary to generate innovation in care and management in addition to recognizing the need for new and better vaccines, designed to be more cost-effective and suitable for low resource settings, for instance, by improving storage conditions and forms of administration.

Additionally, vaccine prices, which have a direct impact on access, are dependent on aspects of intellectual property and production capacity and distribution. Unlike what occurs in the traditional drugs segment, vaccines are biological products that are difficult to reproduce on a large scale and whose production processes may also be subject to patents that impede competition by generic versions at lower prices.

Until the end of the 1970s, vaccine production was based on traditional techniques targeting the pediatric segment, considered as commodities with low profit margins by pharmaceutical companies. With the advent of modern biotechnology and the expansion of development potential with respect to new vaccines to serve different segments of the population, this sector became more attractive to private companies. Interest by Big Pharma intensified beginning around 2000, following the expiration of patents pertaining to bestseller drugs, which resulted in competition from generic drug makers. Registrations of new molecular entities became less frequent and other changes occurred in the pharmaceutical business environment.

These changes in the pharmaceutical industry operational environment, together with the possibilities ushered in by scientific and technological advances, made the vaccine sector highly attractive to major pharmaceutical companies. This also expanded opportunities for the development of preventive and therapeutic vaccines targeting degenerative diseases. In recent decades, the industrial vaccine sector has become highly concentrated and is now dominated by few multinational pharmaceutical companies. Only five of these possess 80% of the worldwide market share [6].

The main vaccine markets in terms of value are located in developed countries, but the biggest demand in terms of volume is located in the rest of world. The vaccine demand in developed countries represented 82% of industry revenue, but this demand is only 12% of its volume [7]. As developing countries tend to consume more traditional vaccines involving lower costs, we believe that the current dynamics of innovation in the vaccine sector tend to further exacerbate inequality with respect to the development and production of vaccines to serve the health needs of populations around the world.

For a long time, R&D to obtain new drugs, vaccines, and other health tools were neglected within the context of public health. Priority was given to health programs and services without considering the allocation of resources towards the development and improvement of new tools. In addition to providing funding for traditional operations and health systems research, as discussed by Moran [8], global health funding must also provide support for new product development by connecting public health and innovation. This author considered R&D funding as an essential aspect of the global effort to reduce infectious disease, as well as child and maternal mortality rates. She also mentioned that, without R&D for new health tools, the Sustainable Development Goals (SDG), which include “ending the epidemics of AIDS, TB, Malaria, and Neglected Tropical Diseases by 2030,” will not be achieved.

From this perspective, beyond the strengthening of health systems and expansion of immunization coverage, there is a need for investment in R&D that enables the obtainment of new vaccines targeting diseases that disproportionately affect poor people and populations living in low- and middle-income countries.

As there is a lack of market incentives for diseases concentrated in poor countries, initiatives have been introduced to address market failures. Some of these are push measures, that is, initiatives directed towards the initial R&D phases that are costly for the public sector and/or nonprofit organizations, while others are pull measures, that is, arrangements focused on markets that offer incentives to private industry to develop products, for example, prolonged patent periods, and priority with respect to “fast-track” testing based on regulatory approval and advance-purchase agreements for the developed products, among other mechanisms [9–13].

Archibugi and Bizzarri (2004) proposed the creation of an international global fund that would directly involve coordination by the public sector in order to overcome market failures. This was designed to incorporate the global medical research agenda to promote preventive immunization targeting pandemics and neglected diseases. These authors advocated this to be the most effective and efficient method of managing international research activities under a formal system of global governance to produce results that would be considered public goods [14]. However, their proposal focuses solely on financial sustainability issues related to R&D and does not address the other challenges faced in vaccine innovation.

In recent years, advance market commitments have been widely touted as an attempt to combine push and pull strategies from the onset to improve the efficiency of markets to accelerate vaccine development as well as facilitate access to new vaccines targeting neglected diseases.

Advance market commitments (AMCs) are generally implemented when new technologies are in the final phases of development as a result of the inherent uncertainty surrounding the initial R&D process, which requires an abundance of time and investment. Meanwhile, the importance of AMC for early-stage products has been defended as an argument to align the incentives in initial stages to increase the chances of obtaining a finalized vaccine product. With respect to private sector interest in these commitments, Kremer et al., 2005, noted that biotech companies and venture capitalists tend to participate in early stages of R&D, while multinational pharmaceutical companies tend to become involved at later stages [15]. Cost estimations for a given set of specified assumptions pertaining to hypothetical vaccine development cases involving advance market vaccine commitment may be sufficient to stimulate research to obtain desired vaccines targeting neglected disease [16].

In the agreements established under AMCs, sponsors commit to subsidizing the future purchase of a vaccine at a certain quantity and a specified price. These types of advance-purchase agreements would accelerate development and access as well as prove cost-effective from a public health perspective, considering the years of life that would be saved [16, 17].

On the other hand, some scholars point out, using empirical evidence and economic analysis, that AMCs could cause serious short-term and long-term complications, including undermining development partnerships. They also claim that no vaccines have been created under these kinds of commitments, since these advance-purchase commitments are actually designed to favor large multinationals and do not recognize the ability of researchers at nonprofit and public research organizations to creatively remove scientific obstacles in the search to discover new effective vaccines. For these scholars, AMCs are indeed not really used to accelerate research but rather to buy extra doses of newly formulated, often cheaper, vaccines that were already previously developed and whose R&D investment was fully recovered from sales in affluent markets. They argue that AMCs need to be designed differently to overcome shortcomings not addressed by push funding from government sources, promoting synergy in research, and mobilizing research teams in all sectors [18].

The process to obtain a new vaccine is specific and complex, requiring some long-term investment strategies. In the biotechnology sector, interinstitutional and interdisciplinary collaboration have been identified as determining factors of innovation by a number of authors. By working in networks, partners become determinants for the competitiveness of companies operating in this sector, and the analysis of these networks constitutes an important step in understanding the innovation dynamic [19–23].

Taking into consideration the fact that this sector is dominated by relatively few pharmaceutical companies that do not invest in R&D for diseases that primarily afflict poor populations with a lack of potentially profitable markets, this article employed an exploratory case study to map out and analyze the types of organizations involved in R&D funding, as well as the progress achieved to date with respect to the pipeline of TB vaccine candidates in clinical trials.

To this end, we believed that the types of organizations involved in funding and the strategies employed to foster vaccine development would provide evidence regarding current efforts to obtain new licensed tuberculosis vaccines, considered one of the most important strategies for the control and prevention of this disease worldwide.

## 2. Case Study: R&D Target to Tuberculosis Vaccines Candidates

Tuberculosis, a chronic and contagious disease, is considered one of the most lethal infectious diseases worldwide [24]. The number of incident cases was approximately 9.6 million in 2014. It is estimated that 1.5 million people died from the disease in 2014, including 400,000 individuals who were also HIV positive [25]. The number of infected people is estimated to be even higher, and evidence suggests that nearly three million cases go undiagnosed or unreported [26]. In general, the symptoms of tuberculosis include coughing, sputum production, appetite loss, weight loss, fever, and night sweats. Initially, the disease affects the lungs, before migrating to other parts of the body, making it a chronic and systemic condition.

Evidence exists indicating that the tuberculosis infection has been with humanity since the Neolithic era [27]. Consumption, phthisis, scrofula, Pott's disease, and the White Plague are all terms that have been used to refer to TB throughout history [28]. Since the early nineteenth century, this disease has been associated with precarious living conditions, particularly malnutrition and overcrowding. In recent decades, new risk factors related to the weakening of individual immune systems have also become associated with the disease [29]. Nonetheless, higher prevalence and incidence continue to affect lower- and middle-income countries, particularly the poorest, most vulnerable populations worldwide. TB has become a stigmatized silent epidemic due to the lack of influence of its carriers.

In recent decades, the HIV/tuberculosis coinfection epidemic, the prevalence of noncommunicable diseases that weaken the immunological system, and the rise and spread of multidrug resistant (MDR) forms of TB have exacerbated this situation. If resistant forms continue to spread and become dominant, the consequences of this disease will be even more serious due to the fact that these forms are not clinically manageable.

Explanations for the inability to attain internationally advocated strategic goals for improving case detection, treatment, or reducing the size of the exposed population include the absence of universal health care to provide access to quality tuberculosis care, as well as a lack of social protection mechanisms in low-income and middle-income countries. Key structural determinants of TB epidemiology include global socioeconomic inequalities [30]. It is estimated that 22 countries, known as TB “high-burden” countries, were responsible for 80% of the world's tuberculosis cases in 2013 and 2014 [24, 31].

Efforts to prevent and control the disease have been based on early diagnosis and rapid onset of treatment to break the chain of transmission. The available medications can be toxic and treatment takes a long time to cure, an average of six months. This extensive time makes abandonment of treatment more likely, increasing chances of developing MDR forms of the disease. In addition, most of the people that are part of the main risk groups are in disadvantage to have access to health care system.

There is just one TB vaccine available, BCG, which was introduced in 1921. It offers partial protection against some forms of pediatric TB but is ineffective against the major burden and lethal form, pulmonary TB. Between the decades of 1920, when the BCG was introduced, and the 1980s, there are no records of research for the development of new tuberculosis vaccines [32].

In 1993, WHO declared the disease a global emergency that had until then been largely neglected, citing the risk that TB could spread with the HIV epidemic, as well as with the rise and spread of multidrug resistant (MDR) forms. Projections by WHO to decrease the rate of tuberculosis deaths of 95% and a 90% incident rate of tuberculosis by 2035 include the licensing of additional tools by 2025 which include a new vaccine [33, 34].

Recognizing the importance and necessity of initiatives to minimize the lack of TB vaccines, we consider it important to inform the public regarding the current situation surrounding the development of new tuberculosis vaccines: who is effectively funding R&D targeting preventive TB vaccines and what are the main strategies being used to speed up the development process?

## 3. Materials and Methods

This is an exploratory case study based on publications and public access databases. The data on financing for research and development of preventive vaccines between 2007 and 2014 have been obtained through the Global Funding of Innovation for Neglected Diseases (G-FINDER) database. G-FINDER is a database that contains only primary data targeted to R&D addressed to neglected diseases. No secondary data or estimates are included. Funders provide data, declaring only funding effectively disbursed. Data and other related information on TB vaccine candidates in clinical trials were obtained from scientific publications and documents and from the sites of involved organizations, as well as the ClinicalTrials.gov database.

## 4. Results and Discussion 

We found 481 records of R&D financing initiatives for TB preventive vaccines between 2007 and 2014 (Table 1), adding up to nearly US$864.2 million for the period (Table 2).

In Table 1, as expected, the public sector and philanthropic institutions are mainly responsible for the initiatives, as pharmaceutical companies do not invest in R&D for diseases that primarily afflict poor populations due to the lack of potentially profitable markets. In general, R&D initiatives aimed at neglected diseases (NDs) are highly expensive for the public sector and/or nonprofit organizations as these attempt to minimize knowledge gaps and market failures, which is also pertinent to Product Development Partnerships (PDPs).

PDPs are initiatives that usually present a hybrid form of the public sector, philanthropic institutions, academia, and private business with the goal of directly advancing to the creation of new products targeted to neglected diseases.

Note that the larger part of private sector initiatives refers to the their own R&D activities (Table 1). The category of self-funding refers to funding that originates within an organization for R&D activities carried out by that organization. While the category of funding intermediaries relates to the organizations that receive funds and disburse them to external product developers, these do not engage in partnerships nor do they actively manage R&D projects [35].

**Figure 1:**
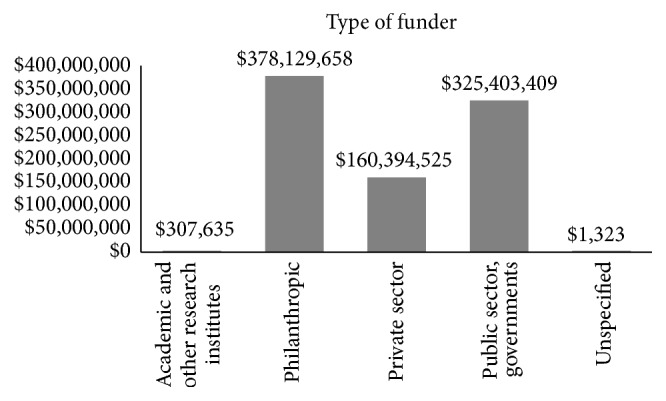
Institutional sources of funding per amount provided (US$). Source: Police Cures. G-FINDER, 2016.

Although most initiatives are carried out by the public sector (Table 1), the majority of resources are supplied by philanthropic organizations (Figure 1).

**Figure 2:**
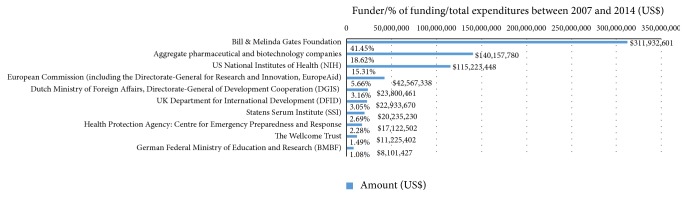
Top 10 TB preventive vaccine funders compromise 95% of total funding. Source: Police Cures. G-FINDER, 2016.

Figure 2 illustrates that the Bill & Melinda Gates Foundation is by far the largest global funder of TB preventive vaccines, followed by aggregate pharmaceutical and biotech companies and the NIH. These funders have different funding models: Bill & Melinda Gates Foundation do not conduct any R&D themselves but provide external funding to other organizations to do so. In the case of pharmaceutical and biotech companies, they use their own budgets to conduct internal R&D programs and they use a self-funded model of participation in PDPs. The NIH, a science and technology agency linked to the USA government, employ a mixed model to provide funding to external organizations but also fund internal R&D programs from their budget [35]. Other actors that have played a key role in funding are aid agencies, multilateral agencies, and domestic science organizations linked to governments.

As shown in Table 2 around 27% of all financing goes to basic research, while the aggregate pharmaceutical and biotechnology companies category receives 20.79% of the total resources. Almost 50% of the total funds go to PDPs. When we disaggregate information regarding who are funding and receiving money in PDPs category (Table 3), we found that the resources are destined for four organizations: Aeras; Infectious Disease Research Institute (IDRI); Tuberculosis Vaccine Institute (TBVI); International Vaccine Institute (IVI).

Intermediaries, such as nonprofit organizations operating PDPs, have been protagonists in the effort to promote conditions favoring the obtainment of technologies to combat neglected disease. They shed light on the specific deficiencies affecting the health of impoverished populations in an attempt to address these concerns and attract financing to their causes. Once resources become available, they initiate projects and specify goals, establish requirements, and divide tasks among the partners. In our view, PDPs are intermediary organizations that act as strategic risk managers, creating niches for specific health products. Their activities include the articulation of expectations and viewpoints to attract attention and resources, as well as providing direction during the learning and technical development processes as discussed by Geels and Raven [36].

There is some discussion regarding the process surrounding brokering and knowledge integration by PDPs to fill private and public organizational gaps that go beyond market failures as a new form of social interaction among different actors to build collective capacity including advocacy to generate political demand for global health products [37, 38].

Of the total R&D funding amount, 49% was allocated to PDPs (Table 2). Of this, as summarized in Table 3, Aeras received 44%, followed by TBVI with 2.5%, IDRI with 2.3%, and finally IVI, which received only 0.2%. Of note is the fact that the Bill & Melinda Gates Foundation is primarily responsible for funding PDPs focused on the technological development of preventative vaccines for TB, as reflected by its share of financing: around 77% of the total funding destined for PDPs.

PDPs are generally nonprofit organizations focused on specific and particular public health issues that drive product development for neglected diseases in conjunction with external partners. The strength of this model is that it brings different actors together to bridge R&D gaps by integrating scientific, technical, and regulatory capabilities under a unified financial and management structure, thereby avoiding the fragmentation of resources and scattered competencies.

Promising research conducted at universities and public research institutions faces challenges in advancing the development of new vaccines due to high cost and the need for specialized capabilities to carry out the steps crucial to achieving licensed technologies. Public-private partnerships, including PDPs, have emerged as an alternative to join nonstate actors and for-profit and nonprofit organizations in less-hierarchical and less-bureaucratic horizontal collaborations, combining government financing and public health priorities with the efficiency and expertise of the private sector [14].

The development and approval of vaccines require rigorous, expensive, and time-consuming testing involving the stages of research, pilot production, and preclinical and clinical testing. The research phase is comprised of steps to discover and select antigens, adjuvants, and delivery methods, setting specific targets, and evaluating immunoprotective responses. Due to the complex and expensive nature of developing new vaccines, PDPs have enjoyed little autonomy with respect to intellectual property rights, manufacturing, marketing, and distribution, which have largely been the responsibility of private companies [39].

A systematic review of qualitative data pertaining to Product Development Partnerships designed to address public health concerns indicated the scarcity of empirical research regarding the functioning of PDPs, specifically with respect to organizational theory. This emphasizes the need to conduct wide-ranging replicable studies aimed at identifying and validating which operational aspects of PDPs lead to successful outcomes [40].

Additionally, some have criticized the overlapping areas of operation among PDPs, the imbalance of influence among member partners, and the lack of transparency in governance structure and agreements pertaining to roles and rewards, including aspects of intellectual property rights favoring biopharmaceutical companies [41].

Vaccines are more regulated than other drugs because they are a biological product, which is, in general, used in healthier people to prevent diseases. Regulatory authorities need to be convinced that a vaccine is safe and efficacious and can be produced within strict standards in a reproducible way. For these reasons, the clinical-trial phase is considered a decisive step in vaccine development process. The results of each clinical trials' phase are considered separately. Even in the most advanced stages of the clinical trials, there is no guarantee of obtaining a licensed vaccine.

In October 2016, we found 16 vaccine candidates that fit our research criteria. After researching, organizing, and refining the information from various sources, we excluded vaccine candidates that were still in the basic research and preclinical phase in order to focus on clinical-trial phase candidates. Of these, nine include the participation of the leading PDP organizations: AERAS, TBVI, and IDRI (Table 4).

The TB vaccines under development are designed to affect various stages of the disease: prevention of initial infection (before exposure); after exposure to prevent the disease after initial infection, latent infection or reactivation from latency and immunotherapy to shorten the course of chemotherapy for active TB and to decrease relapse or reinfection rates that may correlate to latency [42–46]. The strategies are based on distinct technical approaches, operating under the assumption that one approach is not necessarily better than another in light of the fact that a licensed vaccine against pulmonary TB still does not exist. A recent systematic review of publications attempting to mathematically model the epidemiological impact of future vaccines against TB designed to prevent infection, prevent disease, or both concluded that a new TB vaccine would be cost-effective (from both a health system and societal perspective), even in the context of a protection index as low as 20%, especially if delivered to adolescents and adults [47].

The limited understanding of complex immunological mechanisms combined with the highly variable nature of the immune response to the agent that causes tuberculosis, in addition to the lack of appropriate animal models to simulate the natural course of this disease, makes the process of obtaining a new vaccine highly uncertain. The scientific community has widely recognized the present lack of correlates of TB vaccine efficacy as a major bottleneck for the development of improved prophylactic strategies [48, 49]. This lack of correlates of protection involving several immune parameters that are commonly measured in preclinical studies became abundantly clear from the results of the MVA85A efficacy trial, which demonstrated the absence of this strategy's superiority as compared to BCG vaccination alone, despite the magnitude of change observed in several parameters used to measure the adaptive immune response in protocols reporting the development of new candidate vaccines [50]. Furthermore, the correlation of tuberculosis with other risk factors (HIV, malnutrition, diabetes, etc.) increases the complexity of the obstacles that need to be overcome to obtain a secure and effective vaccine [51–54].

The centuries-long relationship between* Mycobacterium tuberculosis* and the human host has shaped the host-pathogen interaction favoring bacilli adaptation to the host's immune system pressure [55]. This constitutes another issue that increases the gaps in knowledge around the immunological system's mechanisms of protection that need to be induced to fight the disease in different groups and populations. It is likely that multiple immune mechanisms would have to be targeted, including pathways nonnaturally induced by mycobacterial challenge [56]. Given the uncertainties associated with obtaining a safe and effective vaccine for tuberculosis, it is impossible to predict the success of vaccine candidates in the short- and medium-terms or the viability of an ideal vaccine intended for all target-populations in a way that attains a strategic global vaccination.

Challenges faced with respect to the sharing of data and accumulated knowledge in the experimental design and research phases are significant, as decisions to design a new vaccine will drive the R&D process and these must be addressed in order to further progress, reduce costs, and shorten the time needed to develop more effective vaccines [57]. Yaqub (2009) called attention to the fact that instruments, skills, and capabilities are critical to the manipulation of conditions that allow a series of measures and controlled experimentation. This permits the resulting knowledge to be tested vis-à-vis specific operational principles based on scientific and technical explanations, which facilitates learning and knowledge accumulation to further technological development. As experimental conditions are created locally their coordination requires shared instrumentalities or standard procedures which must be integrated into the governance of the R&D vaccine research process to make it feasible to systematically accumulate knowledge aimed at innovation [58].

Nelson et al., 2011, argued that the processes of learning and innovation development do not end with the adoption of new technology but rather that these represent the beginning of a continuous and cumulative process that becomes more robust in practice. New scientific knowledge regarding disease is constantly being redesigned according to incremental improvements that rely not only on basic scientific research but also on new advances in technological capabilities originating from different fields that subsidize new diagnostic and therapeutic modalities, as well as learning by doing in clinical practice [59, 60].

Despite the uncertainties inherent in the TB vaccine development process, we found 16 candidates in clinical trials phases, with nine being significantly supported by PDPs (Table 4). This could indicate that the strategies employed by PDPs have enabled more vaccine candidates to enter into the clinical trials phase. Since vaccine candidate clinical trials testing in humans is categorized according to specific patient populations in specific settings, regardless of whether the candidates are promising or not in initial phases, these results represent important sources of information to further the understanding of the complex mechanism by which* M. tuberculosis *evades the immune system and can serve to support new research designed to trigger a unique immune response.

## 5. Conclusions 

Even though global health agencies explicitly state that one of the most pressing priorities concerning public health worldwide is the obtainment of an effective preventive vaccine for TB, more than 90% of the R&D resources allocated to achieve this goal come from a small number of institutions whose financing strategies are not completely aligned with the objectives of coordinated and integrated vaccine development efforts.

Another relevant aspect is that almost half of these resources are destined towards Product Development Partnerships, initiatives that are extremely dependent on the Bill & Melinda Gates Foundation. This highly concentrated dependence on funding originating from the resources of a single organization is concerning, as there is no guarantee regarding the regularity of PDP funding over the medium- or long-term. This preoccupation becomes amplified by the uncertainty involved in obtaining results and outcomes that may not come to light for several years.

Our findings related to organizations involved in clinical trials of vaccine candidates for TB corroborate the notion that the guiding strategies used by PDPs represent the best strategies to integrate and coordinate resources. These aim to ensure that potential products, which lack commercial appeal, are able to advance to the final stages of the clinical trials by enabling complementary scientific, technical, financing, and management capacities, thereby avoiding the disassociation and fragmentation of resources and expertise.

Due to the social relevance of vaccines within the field of public health, it is essential to establish strategic partnerships guided by global health directives via commitments and strategic alignments among governments, the scientific community, and private and philanthropic organizations. The hope is to design and execute strategies and complementary roles in vaccine development, manufacturing, and distribution, including providing guarantees regarding the regularity of global R&D funding to obtain new products targeting neglected diseases. This could overcome the current barriers impeding the development and provision of vaccines for diseases, such as tuberculosis and neglected tropical diseases, which are more prevalent in poor populations and in low- and middle-income countries.

In science-based sectors, such as the vaccine industry, no direct relationship exists between investment and the results produced, as the process of obtaining innovations involves uncertainty. The continuous efforts that characterize the strategies to obtain licensed vaccines are combined and integrated into a specific set of knowledge, skills, and abilities at various stages of development, ranging from the research and discovery of antigens, preclinical and clinical trials, scaling up production, and finally distributing vaccines, all entailing a long and complex journey.

In general, the collaborative networks in the field of neglected diseases take on different roles compared to the alliances formed to develop technologies targeting prevalent diseases in developed countries. Collaborative R&D networks are organizational devices that allow the coordination of heterogeneous learning processes by actors with different skills, competencies, and objectives in which international organizations exert great influence over the coordination of activities.

Because the development of vaccines is more expensive and time-consuming than the process involving drug development, current PDP models working on vaccine development attempt to fill knowledge and R&D funding gaps and are therefore limited with respect to incorporating facets related to the more advanced stages of development that involve regulatory aspects, intellectual property, manufacturing, and distribution. As there is no commercial market to motivate private investment to carry out these kinds of initiatives, models complementary to the current dynamics of development and provision of vaccines must be discussed and implemented in national, global, and political agendas to align industrial policies with health policy.

In this sense, global PDPs can be contemplated to face challenges in the development and provision of vaccines. We believe that the infrastructure needed to produce and distribute vaccines could be part of the PDP model strategy to license vaccines affordably for poor and developing countries, which may involve the active participation of the Developing Countries Vaccine Manufactures Network, which was created with the mission of raising the quality and availability of vaccines in developing countries at affordable prices.

No country can be self-sufficient in the development and production of vaccines, and none attempt to be independent due to the need for economies of scope (diversification) and scale. It is therefore feasible to stimulate strategic selectivity regarding the points of action and cooperation that may generate systemic global institutional arrangements in the process of research, development, licensing, production, and vaccine distribution.

In addition to ethical and humanitarian issues, established, emerging, and reemergent diseases present more than an isolated threat to specific areas and countries, which reinforces the need to develop strategies of global cooperation to obtain and access new vaccines strategies that may be able to reduce the burden of disease and possibly even eradicate it, thusly generating positive social externalities on a global scale.

Despite the growth and importance of PDPs, there has been minimal investigation into the actual governance and learning processes involved in these partnerships which are designed in a network governance structure that reconfigures structural linkages and the role between public and private actors. We believe that future studies will be useful to provide a comprehensive picture of PDPs in an attempt to better understand their governance structures, including the functional operations and decision-making processes pertaining to the design and execution of R&D programs to advance practical recommendations for global PDPs. This would include IP rights, in addition to the planning and implantation of manufacturing and distribution capabilities to deliver licensed products, once they become available, at affordable cost in high-burden countries.

## Figures and Tables

**Table 1:** The nature of the funding organization per funding type provided.

Type of funder	Funding to R&D	Funding to PDPs	Funding to intermediaries	Self-funding	Number of records
Academic and other research institutes	2				2
Philanthropic	51	20			71
Private sector		5	2	20	27
Public sector, governments	300	39	3	38	380
Unspecified	1				1
Total	354	64	5	58	481

Source: Police Cures. G-FINDER, 2016.

**Table 2:** Where does the financing go?

Type of recipient	Amount received (US$)	Percentage of total amount
Product Development Partnerships (PDPs)	425.171.917,91	49,20%
Academic and other research institutions	176.214.720,19	20,39%
Aggregate pharmaceutical and biotechnology companies	179.641.013,81	20,79%
Government research institutions	51.896.955,72	6,00%
N/A	19.216.561,55	2,22%
Other intermediaries	5.208.142,85	0,60%
Private sector philanthropic foundations, trusts, NGOs, corporate donors	2.319.262,62	0,27%
Public sector/governments	4.567.975,11	0,53%
Total	**864.236.549,76**	**100,00**

Source: Police Cures. G-FINDER, 2016.

**Table 3:** PDPs: who is disbursing and receiving funding?

PDPs: who is disbursing and receiving funding?	Amount (US$)	(%)
*Aggregate pharmaceutical and biotechnology companies*		
TuBerculosis Vaccine Initiative (TBVI)	339.951,22	0,04%
*Australian Department of Foreign Affairs and Trade (DFAT), previously the Australian Agency for International Development (AusAID)*		
Aeras	2.480.895,08	0,29%
*Bill & Melinda Gates Foundation*		
Aeras	324.040.619,82	37,49%
TuBerculosis Vaccine Initiative (TBVI)	3.117.718,59	0,36%
*Calouste Gulbenkian Foundation*		
TuBerculosis Vaccine Initiative (TBVI)	208.971,10	0,02%
*Danish Ministry of Foreign Affairs and/or Danish International Development Agency (DANIDA)*		
Aeras	608.710,02	0,07%
*Dutch Ministry of Foreign Affairs, Directorate-General of Development Cooperation (DGIS)*		
Aeras	27.465.410,18	3,18%
*European Commission (including the Directorate-General for Research and Innovation and the Directorate-General for Development and Cooperation, EuropeAid)*		
TuBerculosis Vaccine Initiative (TBVI)	16.260.806,45	1,88%
*Fondation Mérieux*		
TuBerculosis Vaccine Initiative (TBVI)	71.997,60	0,01%
*Institut Mérieux*		
TuBerculosis Vaccine Initiative (TBVI)	202.659,95	0,02%
*National Research Foundation of Korea (NRF)*		
International Vaccine Institute (IVI)	177.367,22	0,02%
*Research Council of Norway*		
Aeras	694.151,99	0,08%
*Royal Norwegian Ministry of Foreign Affairs and/or Norwegian Agency for Development Cooperation (NORAD)*		
TuBerculosis Vaccine Initiative (TBVI)	1.644.524,03	0,19%
*State Government of Maryland*		
Aeras	244.289,61	0,03%
*The Paul G Allen Family Foundation*		
Infectious Disease Research Institute (IDRI)	324.384,65	0,04%
*The Wellcome Trust*		
Infectious Disease Research Institute (IDRI)	1.275.077,75	0,15%
*UK Department for International Development (DFID)*		
Aeras	26.065.268,24	3,02%
*US Centers for Disease Control (CDC)*		
Aeras	243.808,94	0,03%
*US Food and Drug Administration (FDA)*		
Aeras	189.242,32	0,02%
*US National Institutes of Health (NIH)*		
Aeras	865.375,69	0,10%
Infectious Disease Research Institute (IDRI)	18.645.346,30	2,16%
*Wildermuth Memorial Foundation*		
Aeras	5.341,16	0,00%
Total	**425.171.917,91**	**49,20%**

Source: Police Cures. G-FINDER, 2016.

^*∗*^Italic typeface indicates organizations that disburse funding, while all others are recipients.

**Table 4:** Vaccine candidates in clinical trials.

Candidate	Type	Sponsors/partners	Phase
Ad5 Ag85A	Viral vector	McMaster University (Canada); CanSino (China)	I
ID93 + GLA-SE	Adjuvanted subunit	*Infectious Disease Research Institute—IDRI* (USA); *Aeras *(USA)	I
Crucell Ad35/MVA85A	Viral vector	Crucell Holland B.V (Netherlands); University of Oxford (UK); *Aeras* (USA)	I
Dar-901	*M.obuense* whole cell	Dartmouth-Hitchcock Medical Center (USA); *Aeras* (USA)	I
TB/FLU-04L	Viral vector	Research Institute for Biological Safety Problems—RIBSP (Kazakhstan)	I
MVA85A	Viral vector	Oxford University (UK)	I
ChAdOx1-85A/MVA85A	Viral vectors	Oxford University (UK)	I
MVA85A-IMX313	Viral vector	Oxford University (UK); Imaxio (France)	I
MTBVAC	Live genetically attenuated MTB	*Tuberculosis Vaccine Institute—TBVI *(Netherlands); University of Zaragoza (Spain); Biofabri (Spain)	IIa
VPM 1002	Live rBCG	Max Planck Institute; Vakzine Projekt Management GmbH (Germany); *TBVI* (Netherlands); Serum Institute of India (India)	IIa
H1 (Ag85B/ESAT) + IC31	Adjuvanted subunit	Statens Serum Institute—SSI (Denmark ); *TBVI *(Netherlands); Valneva SE (France)	IIa
RUTI (Tb lysate)	Fragmented MTB	Archivel Farma (Spain)	II a
H4 (Ag85B/TB10.4) + IC31	Adjuvanted subunit	Statens Serum Institute—SSI (Denmark); Sanofi-Pasteur (France); *Aeras (*USA); Valneva SE (France)	II a
H56 (Ag85B/ESAT- 6/Rv2660) + IC31	Adjuvanted subunit	Statens Serum Institute—SSI (Denmark); *Aeras (*USA); Valneva SE (France)	II a
M72 + AS01E	Adjuvanted subunit	GSK (UK); *Aeras (*USA)	II b
*M. vaccae*	*M. vaccae* whole cell	Anhui Zhifei Longcom Biologic Pharmacy Co. Ltd. (China)	III

Source: information and data are collected from various sources (2016).
